# Influence of extracellular volume fraction on peak exercise oxygen pulse following thoracic radiotherapy

**DOI:** 10.1186/s40959-021-00127-6

**Published:** 2022-01-18

**Authors:** Justin M. Canada, Elisabeth Weiss, John D. Grizzard, Cory R. Trankle, Leila Rezai Gharai, Franklin Dana, Leo F. Buckley, Salvatore Carbone, Dinesh Kadariya, Anthony Ricco, Jennifer H. Jordan, Ronald K. Evans, Ryan S. Garten, Benjamin W. Van Tassell, W. Gregory Hundley, Antonio Abbate

**Affiliations:** 1 VCU Pauley Heart Center, Department of Internal Medicine, Virginia Commonwealth University, P.O. Box 980335, 1200 E. Broad Street, Richmond, Virginia 23298 USA; 2 Department of Radiation Oncology, Virginia Commonwealth University, Richmond, Virginia USA; 3 Department of Radiology, Virginia Commonwealth University, Richmond, Virginia USA; 4 Department of Pharmacy Services, Brigham and Women’s Hospital, Boston, MA USA; 5 Department of Kinesiology & Health Sciences, College of Humanities & Sciences, Virginia Commonwealth University, Richmond, Virginia USA; 6 Department of Biomedical Engineering, Virginia Commonwealth University, Richmond, Virginia USA; 7 Department of Pharmacotherapy and Outcome Sciences, Virginia Commonwealth University, Richmond, Virginia USA

**Keywords:** Peak exercise oxygen pulse, Extracellular volume fraction, Radiotherapy, Cardiorespiratory fitness

## Abstract

**Background:**

Radiation-induced myocardial fibrosis increases heart failure (HF) risk and is associated with a restrictive cardiomyopathy phenotype. The myocardial extracellular volume fraction (ECVF) using contrast-enhanced cardiac magnetic resonance (CMR) quantifies the extent of fibrosis which, in severe cases, results in a noncompliant left ventricle (LV) with an inability to augment exercise stroke volume (SV). The peak exercise oxygen pulse (O_2_Pulse), a noninvasive surrogate for exercise SV, may provide mechanistic insight into cardiac reserve. The relationship between LV ECVF and O_2_Pulse following thoracic radiotherapy has not been explored.

**Methods:**

Patients who underwent thoracic radiotherapy for chest malignancies with significant incidental heart dose (≥5 Gray (Gy), ≥10% heart) without a pre-cancer treatment history of HF underwent cardiopulmonary exercise testing to determine O_2_Pulse, contrast-enhanced CMR, and N-terminal pro-brain natriuretic peptide (NTproBNP) measurement. Multivariable-analyses were performed to identify factors associated with O_2_Pulse normalized for age/gender/anthropometrics.

**Results:**

Thirty patients (median [IQR] age 63 [57–67] years, 18 [60%] female, 2.0 [0.6–3.8] years post-radiotherapy) were included. The peak VO_2_ was 1376 [1057–1552] mL·min^− 1^, peak HR = 150 [122–164] bpm, resulting in an O_2_Pulse of 9.2 [7.5–10.7] mL/beat or 82 (66–96) % of predicted. The ECVF, LV ejection fraction, heart volume receiving ≥10 Gy, and NTproBNP were independently associated with %O_2_Pulse (*P* < .001).

**Conclusions:**

In patients with prior radiotherapy heart exposure, %-predicted O_2_Pulse is inversely associated markers of diffuse fibrosis (ECVF), ventricular wall stress (NTproBNP), radiotherapy heart dose, and positively related to LV function. Increased LV ECVF may reflect a potential etiology of impaired LV SV reserve in patients receiving thoracic radiotherapy for chest malignancies.

**Supplementary Information:**

The online version contains supplementary material available at 10.1186/s40959-021-00127-6.

## Introduction

Incidental cardiac radiation exposure following anti-cancer thoracic radiotherapy treatment increases risk of heart failure (HF) in a dose-dependent manner [1] with a predominantly restrictive cardiomyopathy phenotype characterized by diffuse fibrosis within the myocardium [2, 3]. The restrictive cardiomyopathy phenotype following radiotherapy is typically a non-infiltrative disorder with endothelial cell damage resulting in microvascular dysfunction and stimulation of excessive extracellular matrix formation leading to increased myocardial fibrosis [4]. This can lead to a noncompliant left ventricle (LV) that is marked by elevated filling pressures and has limited ability to augment stroke volume (SV) [5].

The peak exercise oxygen pulse, determined with cardiopulmonary exercise testing (CPET), is the quotient of oxygen consumption (VO_2_) divided by the heart rate (HR) at peak exercise. Through deduction of the Fick equation, the peak oxygen pulse can accurately serve as a noninvasive estimate of the LV SV response to exercise in both healthy subjects and HF patients [6–9]. An additional noninvasive diagnostic strategy, contrast-enhanced cardiac magnetic resonance (CMR) imaging allows tissue characterization, including quantification of the LV extracellular volume fraction (LV ECVF), a surrogate of diffuse myocardial fibrosis [10]. Knowledge of the relationship between LV ECVF and the peak exercise oxygen pulse may provide mechanistic insight into the cardiac reserve of the cancer survivor following thoracic radiotherapy. The purpose of the current study was to examine this relationship in a cross-section of patients with a history of this treatment.

## Methods

This study complies with the Declaration of Helsinki, was approved by the Virginia Commonwealth University (VCU) Massey Cancer Center Protocol Review and Monitoring Committee and Institutional Review Board, and all subjects provided informed consent prior to enrollment.

### Patients

Patients with a history of external-beam thoracic radiotherapy for the treatment of chest malignancies with significant incidental heart exposure defined as ≥5 Gray (Gy) to ≥10% of the heart volume but were without a pre-cancer treatment history of overt cardiovascular disease (CVD) or HF, were prospectively enrolled. Subjects underwent CPET to determine cardiorespiratory fitness (CRF) including the peak oxygen pulse, N-terminal pro-brain natriuretic peptide (NTproBNP) measurement as a marker of ventricular wall stress [11], and contrast-enhanced CMR imaging. Exclusion criteria consisted of contraindications to CMR with gadolinium-contrast use, moderate or severe renal impairment (glomerular filtration rate < 60 mL/min/1.73 m^2^), pregnancy, or inability to walk on a treadmill.

Clinical variables analyzed were age, sex, race (Caucasian/African-American), history of established CVD risk factors (Yes/No; hypertension, diabetes mellitus, current cigarette smoking, hypercholesterolemia, obesity [body mass index (BMI) ≥30]), cancer type (breast/ lung or other chest malignancy), prior chemotherapy including type and dose, presence of anemia, and current beta-blocker and/or angiotensin-converting enzyme inhibitor (ACEI)/ angiotensin receptor blockers (ARB) defined per medical record review and patient interview. Due to the likelihood of coexistent chronic obstructive pulmonary disease (COPD) in chest tumor patients the presence of COPD was also considered based upon Global Initiative for Chronic Obstructive Lung Disease criteria from pre-exercise spirometry [12]. Physical activity participation was quantified using a validated questionnaire [13]. Quality of life (QOL) was analyzed using the Functional Assessment of Cancer Therapy-General (FACT-G7) questionnaire [14]. Some individuals in this cohort have previously been partially characterized [15].

### Radiotherapy parameters

Radiation dose calculation was performed based on a volumetric computed-tomography (CT) data set obtained during the pre-radiotherapy treatment planning session. A single radiation oncologist (E.W.) performed quantification of total radiation dose and heart volumes exposed. The heart was manually contoured on each CT-slice generating 3-dimensional structures using treatment planning software (Pinnacle, Koninklijke Philips N.V.). After radiation beam definition and target dose calculation, mean cardiac radiation dose (MCRD) was determined for the whole organ volume as well as using dose-volume histograms to generate %volumes (V) of the heart receiving ≥5, 10, 20, 30, 40, and 50 Gray (Gy), respectively.

### Cardiopulmonary exercise testing

A symptom-limited CPET was administered using a conservative ramping treadmill protocol according to established guidelines [16, 17]. The average value for VO_2_ obtained during the final 30-s of exercise was used to define peak VO_2_. The peak exercise respiratory exchange ratio (RER) was defined as the ratio between the peak carbon dioxide production divided by the peak VO_2_. Predicted peak VO_2_ was calculated according to the reference equations proposed by Wasserman and colleagues which take into account age, gender, and anthropometric (height, bodyweight) differences [7]. The absolute peak exercise oxygen pulse (milliliters [mL] of O_2_ per heart beat) was determined by dividing the absolute peak VO_2_ (mL/minute) by the HR at peak exercise. The predicted peak exercise oxygen pulse was calculated as the quotient of the predicted peak VO_2_ divided by the age-predicted maximal HR (220 – age). The peak oxygen pulse was normalized as a percent-predicted value (%O_2_Pulse) to account for subject differences due to age, sex, and anthropometrics. Heart rate (via 12-lead electrocardiography; GE Healthcare, Chicago, IL), blood pressure ([BP], via automated-stress BP monitor; Tango+, Morrisville, NC), and peripheral oxygen saturation ([SpO_2_]; Nellcor, Minneapolis, MN) were assessed according to standard recommendations [16].

### Cardiac magnetic resonance

Resting cardiac magnetic resonance imaging was performed on a Siemens Aera 1.5 Tesla scanner (Siemens Healthcare, Erlangen, Germany). All CMR parameters were interpreted by board-certified cardiovascular radiologists (J.G., L.R-G., F.D.) who were blinded to the results of the remainder of study procedures. Selected MRI sequences were obtained including cardiac dimensions, systolic function, and late-gadolinium enhancement (LGE) post-contrast images. Contrast was administered using 0.2 mmol/kg of intravenous Prohance (Bracco Diagnostics Inc., Monroe Township, NJ). The myocardial volumes; left-ventricular end-diastolic volume (LVEDV), left-ventricular end-systolic volume (LVESV), stroke volume (SV), and cardiac output (CO) were indexed to body surface area. Myocardial tissue composition was quantified through the measurement of native T1 and post-contrast gadolinium-enhanced T1 values using a balanced steady-state free-procession modified Look-Locker inversion recovery pulse sequence. Post-contrast images were obtained at 15-min following contrast administration. Estimation of the ECVF was used to quantify diffuse myocardial injury. The ECVF was determined by the equation [18]:.

$$\mathrm{ECV}=\left(1-\mathrm{hematocrit}\right)\frac{\frac{1}{\mathrm{post}-\mathrm{contrast}\kern5.0pt \mathrm{T}1\kern5.0pt \mathrm{myo}}-\frac{1}{\mathrm{native}\kern5.0pt \mathrm{T}1\kern5.0pt \mathrm{myo}}}{\frac{1}{\mathrm{post}-\mathrm{contrast}\kern5.0pt \mathrm{T}1\kern5.0pt \mathrm{blood}}-\frac{1}{\mathrm{native}\kern5.0pt \mathrm{T}1\kern5.0pt \mathrm{blood}}}$$

**Fig. 1:**
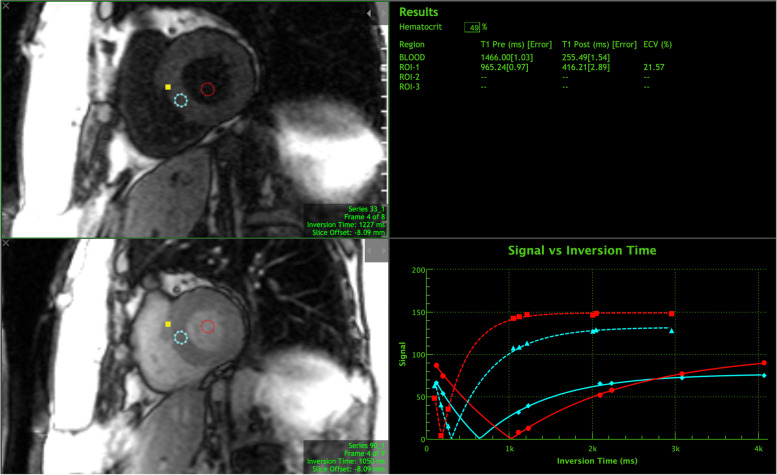
Example of myocardial ECVF calculation from native T1, post-contrast T1, and blood pool inversion times. Abbreviations: ROI = region of interest; ECVF = extracellular volume fraction; ms = milliseconds

A global ECVF was determined from regions of interest in the septum on short-axis slices obtained at the base, mid, and apex of the LV myocardium. Figure 1 represents an example of an ECVF calculation. Hematocrit used in the ECVF calculations was determined non-invasively from the blood pool using a validated technique [19, 20]. Post-processing was performed using dedicated CMR analysis software (Precession, Heart Imaging Technologies, Durham, NC).

### Statistical analysis

Data are reported as number (%) or median [interquartile range] due to potential non-Gaussian distributions. Chi-square tests were performed to assess differences between nominal variables including Fisher’s exact test for variables with cell count frequencies < 5. Spearman’s rank test was used for univariate analysis of associations between continuous clinical characteristics, radiotherapy, CMR, and CRF variables. The Mann-Whitney U test was performed to compare differences between groups based upon categorical variables and peak oxygen pulse values < 85% or ≥ 85% of predicted [21] and LV ECVF values > or ≤ the median value. A threshold value of < 85% of predicted for peak oxygen pulse values has previously been shown to confer increased risk of cardiac mortality in HF patients [21]. A block multivariate linear regression model was created by first evaluating significant nominal categorical predictors of the %O_2_Pulse (block-1) followed by inclusion into a stepwise multivariate model combined with significant continuous univariate radiotherapy and CMR variables to determine predictors of %O_2_Pulse (block-2). All regression beta-coefficients are reported as standardized values. Collinearity diagnostics were performed on significant univariate predictors before entry into the multivariate analysis. Statistical analysis was performed using SPSS v26.0 (IBM Corp, Armonk, NY) with significance set at a *P* < 0.05.

## Results

### Patient characteristics

Thirty patients (age 63 [57–67] years, 18 [60%] female, 2.0 [0.6–3.8] years since completion of radiotherapy) underwent evaluation. The peak VO_2_ was 1376 (1057–1552) mL·min^− 1^ with a peak HR of 150 (122–164) bpm resulting in a peak exercise O_2_ pulse of 9.2 (7.5–10.7) mL/beat that was 82 (66–96)% of predicted values. Table 1 provides the detailed clinical characteristics of the cohort including grouped comparisons between those with a peak exercise oxygen pulse < 85% or ≥ 85% of predicted. Sixteen (53%) subjects demonstrated a peak exercise oxygen pulse < 85% of predicted values. When the peak exercise %O_2_Pulse was evaluated as a continuous variable according to nominal clinical characteristics there were significant differences with respect to smoking status, diabetes, obesity, cancer type, COPD, ACEI/ARB use, and sex. There was not a significant difference between %O_2_Pulse and race, hypercholesterolemia, hypertension, anemia, beta-blocker use, or prior receipt of chemotherapy.

**Table 1:** Clinical Characteristics of the Cohort

Variables	Entire Cohort *N* = 30	O_2_ Pulse < 85% *n* = 16	O_2_ Pulse ≥85% *n* = 14	*P*-Value
Age, y	63 [57–67]	61 [54–64]	64 [58–69]	0.249
Female	18 (60%)	7 (44%)	10 (71%)	0.076
Race				0.107
Caucasian	20 (67%)	9 (56%)	5 (36%)	
African-American	10 (33%)	7 (44%)	2 (14%)	
Body Mass Index, kg/m^2^	27.1 [23.6–30.6]	24.5 [20.8–27.2]	30.3 [27.0–31.2]	0.005
Body Surface Area, m^2^	1.88 [1.73–1.99]	1.81 [1.71–1.94]	1.92 [1.79–2.14]	0.132
COPD	18 (60%)	13 (81%)	5 (36%)	0.023
FACT-G7 score	20.0 [15.0–23.5]	17.5 [14.3–22.3]	21.0 [18.0–25.0]	0.041
Cancer type				0.008
Lung or Other*	20 (67%)	14 (88%)	5 (36%)	
Breast	10 (33%)	2 (13%)	8 (57%)	
Other Malignancies				
Esophageal	2 (7%)			
Hodgkin’s Lymphoma	1 (3%)			
Desmoid Tumor	1 (3%)			
Castleman’s Disease	1 (3%)			
Time since Diagnosis, y	2.6 [1.3–4.2]	2.8 [1.4–7.0]	2.4 [0.6–2.8]	0.398
Time since Radiotherapy, y	2.0 [0.6–3.8]	2.2 [0.8–5.8]	2.2 [0.6–2.8]	0.423
Time since chemotherapy, y	1.7 [0.5–2.9]	1.5 [0.2–4.2]	1.9 [0.6–2.8]	0.423
Prior Chemotherapy	26 (87%)	14 (88%)	11 (79%)	0.617
CVD Risk Factors				
Hypertension	17 (57%)	8 (50%)	8 (57%)	0.404
Diabetes Mellitus	7 (23%)	1 (6%)	6 (43%)	0.019
Hypercholesterolemia	14 (47%)	8 (50%)	6 (43%)	0.566
Current Smoker	6 (20%)	5 (31%)	1 (7%)	0.037
Obesity	10 (33%)	3 (19%)	7 (50%)	0.064
Beta-blocker Use	5 (17%)	2 (13%)	3 (21%)	0.396
ACEI/ARB Use	6 (20%)	2 (13%)	4 (29%)	0.423
NTproBNP, pg/mL	187 [51–310]	298 [89–445]	72 [31–200]	0.013

Data are listed as median and [interquartile range] or n (%). *Other malignancies grouped with lung cancer subjects. *P*-values are differences between groups (< 85% and ≥ 85% predicted peak exercise O_2_ Pulse)

*Abbreviations*: *y* years, *COPD* chronic obstructive pulmonary disease, *FACT-G7* Functional Assessment of Cancer Therapy-General (7-item version), *ACEI/ARB* angiotensin converting enzyme inhibitor/angiotensin receptor blocker, *NTproBNP* N-terminal pro-brain natriuretic peptide

Table 2 provides a detailed description of selected CPET variables. Overall, the peak VO_2_ was moderately-reduced (62% of predicted) compared to normalized values. The peak exercise %O_2_pulse demonstrated a significant positive correlation with the peak VO_2_ (*r* = + 0.78, *P* < 0.001) and a significant inverse relationship with the cardiac biomarker NTproBNP (*r* = − 0.51, *P* = 0.004). The peak %O_2_Pulse and peak VO_2_ were positively associated with FACT-G7 scores (*r* = + 0.39, *P* = 0.038; *r* = + 0.40, *P* = 0.031, respectively) reflecting higher CRF correlated with higher QOL.

**Table 2:** Cardiopulmonary Exercise Test Variables

Variables	Entire Cohort	O_2_ Pulse < 85%	O_2_ Pulse ≥85%	*P*-Value
Peak VO_2_, mL·min^− 1^	1376 [1057–1552]	1166 [805–1330]	1575 [1439–1946]	< 0.001
Percent-predicted peak VO_2_, %	62 [52–89]	53 [46–61]	91 [69–96]	< 0.001
Peak VO_2_, mL·kg^− 1^·min^− 1^	16.9 [14.4–20.8]	16.1 [12.4–18.2]	19.7 [16.5–22.8]	0.012
Peak O_2_ Pulse, mL·beat^− 1^	9.2 [7.5–10.7]	7.9 [6.9–9.3]	10.4 [9.3–12.5]	0.001
%O_2_Pulse	82 [66–96]	68 [55–76]	98 [93–112]	< 0.001
Peak RER	1.02 [0.95–1.09]	0.97 [0.92–1.09]	1.05 [1.00–1.11]	0.045
Resting Heart Rate, bpm	73 [68–86]	77 [67–86]	73 [66–76]	0.449
Exercise Heart Rate, bpm	150 [122–164]	136 [115–157]	151 [141–170]	0.101
Rest Systolic BP, mmHg	124 [111–143]	130 [113–144]	123 [103–140]	0.531
Rest Diastolic BP, mmHg	70 [63–81]	70 [67–82]	68 [57–79]	0.374
Exercise Systolic BP, mmHg	174 [155–190]	175 [150–182]	170 [158–201]	0.475
Exercise Diastolic BP, mmHg	70 [70–80]	72 [70–80]	70 [63–81]	0.329
Breathing Reserve, %	33 [22–46]	24 [16–44]	37 [26–49]	0.156
Peak SpO_2_, %	97 [95–99]	98 [94–100]	97 [96–99]	0.682

Data are listed as median and (interquartile range). *P*-values are differences between groups (< 85% and ≥ 85% predicted peak exercise O_2_ Pulse)

*Abbreviations*: *VO*_*2*_ oxygen consumption, *O*_*2*_ oxygen, *%O*_*2*_*Pulse* percent-predicted peak exercise oxygen pulse, *RER* respiratory exchange ratio, *BP* blood pressure, *SpO*_*2*_ peripheral oxygen saturation

The peak exercise %O_2_Pulse was not significantly associated with age, physical activity levels, rest or maximal HR or blood pressures. From a chronicity perspective, there was not a significant association between %O_2_Pulse achieved and time from cancer diagnosis or completion of chemotherapy or radiotherapy.

### Cardiac magnetic resonance imaging

**Fig. 2:**
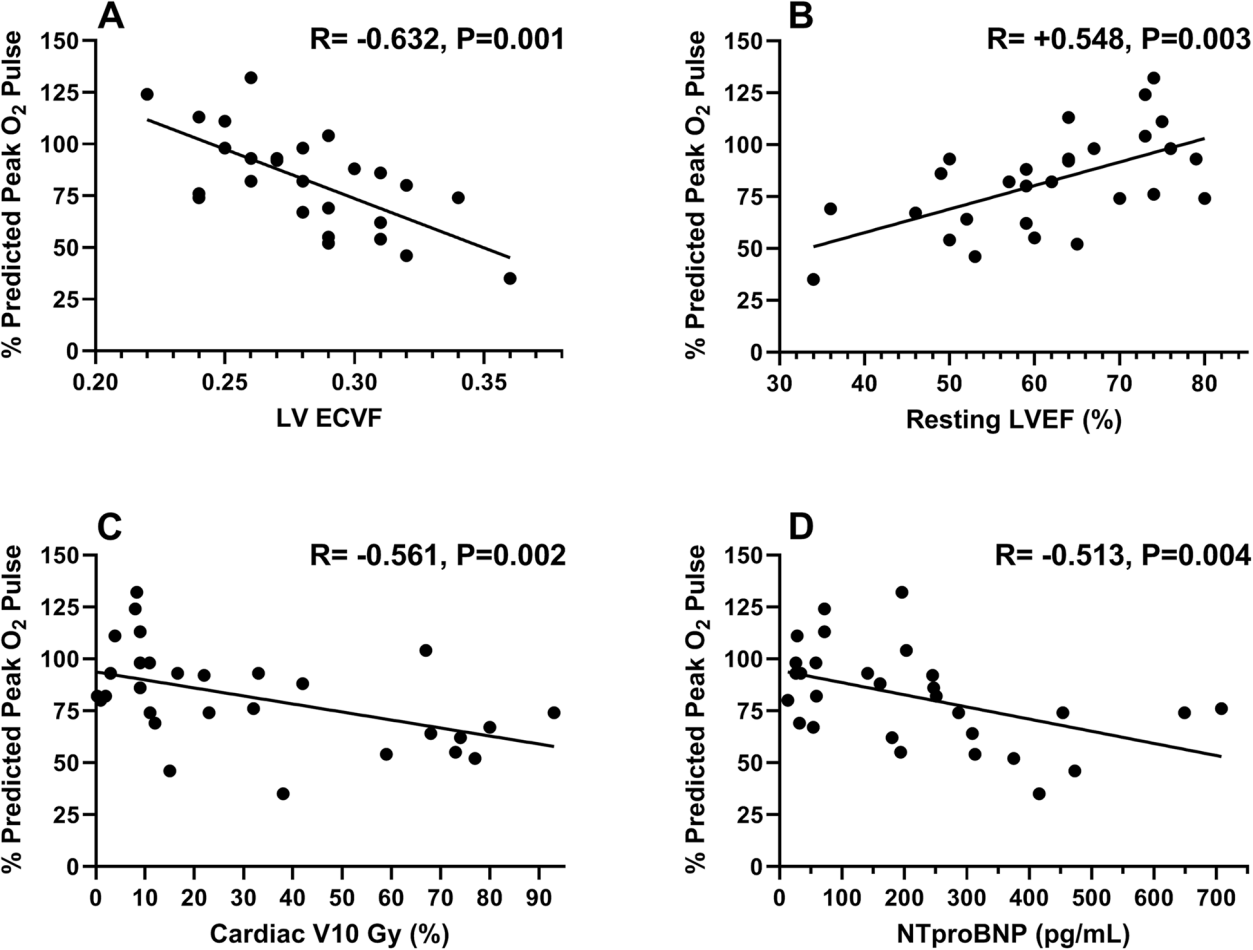
Independent predictors of peak exercise %O_2_ pulse. **Legend: Panel A** - correlation between LV ECVF and %O_2_ pulse. **Panel B -** correlation between resting LVEF and %O_2_ pulse. **Panel C** - correlation between cardiac V10Gy and %O_2_ pulse. **Panel D** shows the inverse correlation between NTproBNP and %O_2_ pulse. Abbreviations: %O_2_ pulse = %-predicted oxygen pulse; ECVF = extracellular volume fraction; LVEF = left-ventricular ejection fraction; V10Gy = %volume of the heart receiving ≥10 Gray; NTproBNP=N-terminal pro-brain natriuretic peptide

Cardiac magnetic resonance variables of interest are detailed in Table 3. Most patients had left-ventricular ejection fractions (LVEF) within normal range (64 [53–74]%), with nearly half (*n* = 12 [41%]) of subjects exhibiting qualitative evidence of LGE with post-contrast CMR. Analysis of CMR variables with peak exercise %O_2_Pulse demonstrated a negative correlation with the composite LV ECVF (*r* = − 0.63, *P* = 0.001), a positive correlation with LVEF (*r* = + 0.55, *P* = 0.003) (Fig. 2; Panels A,B) and the resting SV index (*r* = + 0.52, *P* = 0.011). There was no significant correlation between the %O_2_Pulse and native T1 (*r* = − 0.23, *P* = 0.269), post-contrast T1 (*r* = − 0.20, *P* = 0.340), or %LGE burden (*r* = − 0.03, *P* = 0.878). Similarly, there was no significant difference between dichotomous qualitative presence of LGE and %O_2_Pulse (*P* = 0.99).

**Table 3:** Cardiac Magnetic Resonance Imaging Parameters

Variables	Entire Cohort *N* = 27	Peak O_2_ Pulse < 85% *n* = 12	Peak O_2_ Pulse ≥85% *n* = 15	*P*-Value
LVEF, %	64 [53–74]	59 [50–65]	67 [62–75]	0.046
LVEDV Index, mL/m^2^	62 [55–71]	61 [49–71]	62 [56–74]	0.651
LVESV Index, mL/m^2^	25 [18–32]	24 [18–38]	25 [15–32]	0.695
LV SV Index, mL/m^2^	38 [31–44]	32 [27–42]	39 [34–45]	0.044
LV Cardiac Index, L·min^−1^/m^2^	2.7 [2.2–3.0]	2.3 [1.8–3.0]	2.7 [2.6–2.9]	0.190
Presence of LGE	12 (44%)	5 (42%)	7 (47%)	0.274
LGE, %	0 [0–3]	0 [0–2]	2 [0–4]	0.427
Native T1-Global, ms	1042 [1013–1063]	1044 [1019–1070]	1039 [1009–1047]	0.631
Post-contrast T1-Global, ms	434 [410–463]	425 [386–473]	436 [416–454]	0.781
LV ECVF-Global, %	28 [26–31]	29 [28–32]	27 [25–29]	0.036

Data are listed as median and [interquartile range] or n (%). *P*-values are differences between groups (< 85% and ≥ 85% predicted peak exercise O_2_ Pulse)

*Abbreviations*: *LVEF* left-ventricular ejection fraction, *LVEDV* left-ventricular end-diastolic volume, *LVESV* left-ventricular end-systolic volume, *SV* stroke volume, *LGE* late-gadolinium enhancement, *T1* myocardial T1, *LV ECVF* left-ventricular extracellular volume fraction

The global LV ECVF demonstrated significant relationships with LVEF (*r* = − 0.51, *P* = 0.006), peak VO_2_ (*r* = − 0.42, *P* = 0.032), cancer type (higher in lung or other chest malignancy vs. breast cancer; *P* = 0.020) and use of angiotensin blockade (lower in patients on ACE-I/ARB; *P* = 0.007) (Supplemental Fig. 1). The LV ECVF did not significantly associate with presence of established CVD risk factors (all *P* > 0.050), MCRD or V5-50Gy heart doses (all *P* > 0.050), age (*P* > 0.136), presence of COPD (*P* = 0.190), or prior chemotherapy use (*P* = 0.278).

### Radiotherapy Dosimetric and chemotherapy variables

The total prescribed radiotherapy dose was 60.0 (48.0–60.4) Gy, MCRD 5.6 (3.7–17.8) Gy, and the %-volume of heart exposed to V5Gy was 39.5 (15.8–80.5)%, V10Gy 19.3 (8.8–67.3)%, V20Gy 7.0 (1.2–35)%, V30Gy 2.5 (0–15)%, V40Gy 1.0 (0–7.8)%, and V50Gy 0 (0–3)%, respectively. There was a negative correlation between the MCRD and the peak %O_2_Pulse (*r* = − 0.51, *P* = 0.005). Additionally, all %-volumes of the heart receiving between V5-V50Gy of radiotherapy demonstrated a negative association with peak %O_2_Pulse (V5Gy [*r* = − 0.51, *P* = 0.004], V10Gy [*r* = − 0.56, *P* = 0.002], V20Gy [*r* = − 0.45, *P* = 0.014], V30Gy [*r* = − 0.48, *P* = 0.009], V40Gy [*r* = − 0.54, *P* = 0.003], V50Gy [*r* = − 0.42, *P* = 0.023], respectively). Total prescribed radiotherapy dose was not associated with %O_2_Pulse (*r* = − 0.06, *P* = 0.761).

Due to the heterogeneity of chemotherapy use, comparison with peak %O_2_Pulse was restricted to those chemotherapy agents wherein at least 20% of the cohort received that agent. The doses of the chemotherapy agents analyzed included paclitaxel (*r* = + 0.42, *P* = 0.090), carboplatin (*r* = + 0.38, *P* = 0.226), cyclophosphamide (*r* = + 0.12, *P* = 0.774), and doxorubicin (*r* = − 0.40, *P* = 0.313), which were not significantly associated with peak %O_2_Pulse. Supplemental Table 1 provides the chemotherapy regimens of the cohort.

### Peak oxygen pulse & LV ECVF threshold comparisons

**Fig. 3:**
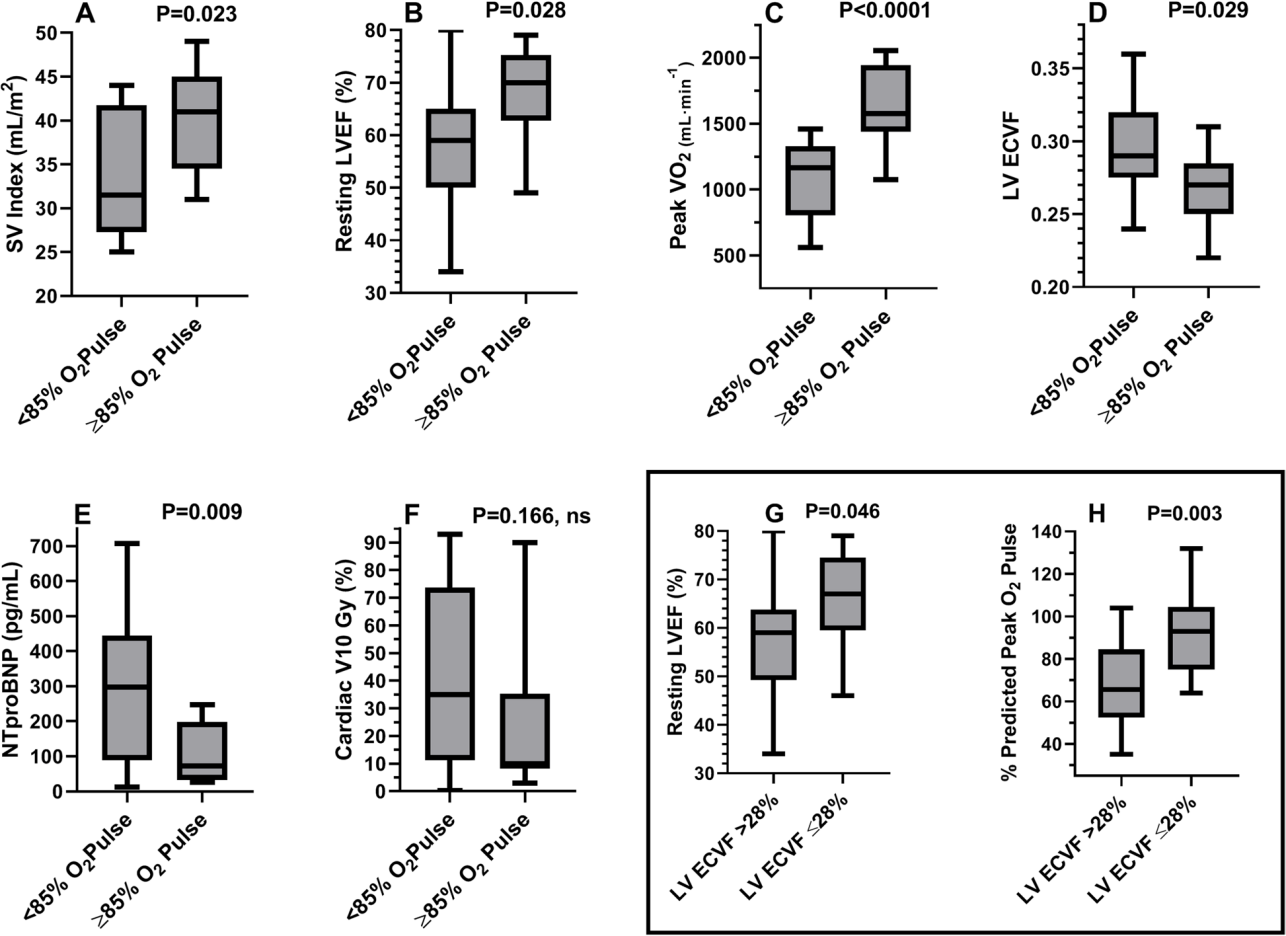
Differences between groups based upon percent-predicted peak exercise O_2_ pulse or median LV ECVF. **Legend:** Box and whisker plots (**Panels A-F**) demonstrating median (horizontal line within rectangular box), interquartile range, whiskers, and range between groups based upon %O_2_ pulse (< or ≥ 85%). **Panel A**: Lower SVI in those with peak O_2_ pulse < 85% of predicted. **Panel B**: Lower LVEF in those with a peak O_2_ pulse < 85% of predicted. **Panel C**: Lower peak VO_2_ in those with a peak O_2_ pulse < 85% of predicted. **Panel D**: Higher LV ECVF in those with a peak O_2_ pulse < 85% of predicted. **Panel E**: Higher NTproBNP levels in those with a peak O_2_ pulse < 85% of predicted. **Panel F**: Trend towards higher V10Gy in those with a peak O_2_ pulse < 85% of predicted. **Panel G**: Lower resting LVEF in those with LV ECVF values >median. **Panel H**: Lower peak %O_2_ pulse values in those with LV ECVF >median. Abbreviations: SVI = stroke volume index; O_2_ = oxygen; LVEF = left-ventricular ejection fraction; VO_2_ = oxygen consumption; ECVF = extracellular volume fraction; NTproBNP=N-terminal pro-brain natriuretic peptide; V10Gy = %volume of heart receiving ≥10 Gray

When dichotomized based upon a peak oxygen pulse < or ≥ 85% of predicted values, a known prognostic threshold [21], there were significant group differences between the SV index, LVEF, peak VO_2_, LV ECVF, and NTproBNP (Fig. 3; Tables 1-3). Furthermore, separation of the LV ECVF at the median value (≤ or > 28%) revealed a significant difference between the LVEF and %O_2_Pulse (Fig. 3; Panels G, H). Supplemental Table 2 provides a comparison of groups separated by the LV ECVF ≤28% or > 28% of the median value.

### Multivariate analysis model

In the multivariate model, significant nominal-level clinical predictors of %O_2_Pulse analyzed in block-1 included: smoking status, diabetes, obesity, sex, cancer type, ACEI/ARB use, and COPD. Cancer type was the only nominal variable retained (*β* = .644, *P* < 0.001) in block-1 of the multivariate model with an adjusted-*R*^2^ of 0.394.

When evaluating radiotherapy dose parameters, collinearity diagnostics revealed significant multicollinearity between the MCRD and V5-V50Gy doses. Due to this finding, the cardiac V10Gy was the only radiotherapy variable entered into the multivariate model as it had the highest univariate relationship with %O_2_Pulse.

In block-2 of the multivariate model, cancer type (block-1) was added to the significant univariate continuous predictors: LV ECVF, LVEF, cardiac V10Gy, and NTproBNP into a stepwise analysis. The LV ECVF (*β* = −.281, *P* = 0.049), LVEF (*β* = .455, *P* = 0.002), cardiac V10Gy (*β* = −.330, *P* = 0.006), and NTproBNP (*β* = −.319, *P* = 0.013) were retained in the model as independent predictors of %O_2_Pulse while cancer type was removed from the model. The overall-model fit demonstrated an adjusted-*R*^2^ of 0.732.

## Discussion

In this cross-sectional analysis of patients with significant incidental heart exposure following thoracic radiotherapy without an established history of CVD or HF, we found peak exercise %O_2_Pulse (a normalized surrogate of exercise LV SV response) was inversely associated with a CMR-derived marker of diffuse myocardial fibrosis (LV ECVF), ventricular wall stress (NTproBNP), cardiac radiation dose, and positively related to cardiac function (LVEF). This demonstrates a potential mechanistic relationship between the effects of thoracic radiotherapy on myocardial function and tissue composition (evidenced by increased LV ECVF, decreased LVEF, elevated NTproBNP) and its resulting influence on cardiac reserve measured via peak exercise %O_2_Pulse. Although the adverse effects of thoracic radiotherapy on late CVD/HF risk are well-known optimal methods for screening to detect subclinical cardiac dysfunction and the role of advanced imaging or exercise testing remain understudied [22, 23].

We recently observed reductions in peak VO_2_ following thoracic radiotherapy driven primarily by impaired diastolic reserve [15, 24]. Increased interstitial myocardial fibrosis that can occur following cancer treatment can impair LV systolic and diastolic function [25]. Work has demonstrated elevated LV ECVF in anthracycline-treated patients (29% received chest radiation) that was negatively associated with diastolic function with lower diastolic function in those with concomitant reduced systolic function [26]. Takagi and colleagues prospectively evaluated changes in global LV function and myocardial tissue characterization in 24 esophageal cancer patients following chemo-radiation therapy which demonstrated early changes in LV ECVF and native T1 (increased 0.5-years after) followed by late (1.5-years after) adverse changes in the SV index [27]. Although CRF was not evaluated it highlights the ability of LV ECVF to detect early subclinical changes that translate into later functional decline (reduced SV). In childhood cancer survivors that previously underwent anthracycline-based chemotherapy regimens (17% received chest irradiation), Tham et al. demonstrated an inverse relationship between LV ECVF and peak VO_2_, however, oxygen pulse was not reported [28]. Similarly, Duca et al. showed an inverse association between LV ECVF and functional status (6-min walk distance, New York Heart Association class) and stroke volume in patients with HF and preserved ejection fraction (HFpEF) [29]. In a prospective study of the ability to differentiate hypertensive heart disease and HFpEF, Mordi et al. demonstrated LV ECVF was an excellent discriminator between hypertension and HFpEF (area under the curve = 0.88) and was inversely associated with peak VO_2_ [30].

The peak oxygen pulse is an expression of peak VO_2_ that by its nature corrects for HR and when further corrected for age, sex, and anthropometrics can differentiate cardiac from noncardiac causes of exercise intolerance [31, 32]. The findings of the current study have implications, as the peak O_2_ pulse is associated with risk for sudden cardiac death, fatal coronary heart disease, CVD, and all-cause mortality [33–35]. Moreover, the peak %O_2_Pulse provides additive predictive accuracy to intermediate-range peak VO_2_ (10–14 mL·kg^− 1^·min^− 1^) for mortality risk assessment in HF patients [21]; some reports have even suggested superiority of the peak O_2_ pulse over peak VO_2_ for predicting clinical HF events particularly when it’s normalized for body mass [36]. Although not reporting oxygen pulse, in a retrospective analysis of patient with cancer therapy-induced HF (CTHF) (44% received chest radiotherapy) compared with non-cancer therapy HF (NCTHF), CTHF patients demonstrated a distinct profile of higher LVEF and worse LV diastolic and systolic (global longitudinal strain) function that was associated with a lower peak VO_2_ and higher incidence of the composite endpoint (all-cause mortality, heart transplant or LV assist device implant) [23]. Furthermore, in an adjusted multivariate analysis (including peak VO_2_, VE/VCO_2_ slope) CTHF was associated with a higher risk of death and the composite endpoint.

In oncological surgery patients, an abnormal exercise oxygen pulse response has been associated with increased mortality, neoadjuvant treatments, and was a strong independent predictor of post-operative cardiopulmonary complications even surpassing peak VO_2_ [37–39]. Collectively, these findings suggest a potential role of CPET including measurement of oxygen pulse in the risk stratification and determination of the causes of exercise intolerance in cancer survivors including those receiving thoracic radiotherapy. To the best of our knowledge, we are not aware of any prior studies that have examined the association between the peak exercise oxygen pulse and LV ECVF.

### Study limitations

The limitations of this study are its small-sample size, single-center, and cross-sectional nature, limiting the findings to hypothesis generating rather than definitively establishing causality and require further exploration. Another limitation is that evaluation of the oxygen pulse is complex, as it depends upon many factors such as fitness, body mass, and is also significantly influenced by arterial oxygen supply and peripheral oxygen extraction. However, in the current study normalization of the oxygen pulse reported as a %-predicted value accounted for the influences of age, sex, and anthropometrics. Furthermore, arterial oxygen supply (quantified by SpO_2_), presence of anemia, or COPD were not significant predictors of %O_2_Pulse in the current study. Unfortunately, the influence of peripheral oxygen extraction was not directly assessed in the current study. Future studies should explore the relationships of radiotherapy dose to specific cardiac substructures with %O_2_Pulse and LV ECVF for a more precise mechanistic understanding of radiotherapy-related cardiac dysfunction [40]. Lastly, the synthetic hematocrit used for the derivation of LV ECVF was analyzed using an automated method based on the linear relationship between the longitudinal T1 relaxation properties of the blood and blood hematocrit versus direct assessment.

## Conclusions

A multimodality noninvasive assessment, including myocardial tissue composition (using contrast-enhanced CMR to derive LV ECVF) and assessment of cardiac reserve (using CPET to determine the normalized peak exercise %O_2_Pulse) may provide mechanistic insight into the causes of exercise intolerance in the cancer survivor following thoracic radiotherapy, suggesting that an expansion of myocardial LV ECV may be contributing to the inability to increase LVSV during exercise leading to reductions in exercise capacity. Although requiring further study, our findings indicate reductions in %O_2_Pulse may represent subclinical reductions in stroke volume that is associated with elevations in LV ECVF. This may allow early detection of radiotherapy-related myocardial dysfunction prior to the onset of overt HF thus opening the door to prophylactic therapeutic interventions.

## Supplementary Information

**Additional file 1.**

**Additional file 2.**

**Additional file 3.**

## Data Availability

The dataset analyzed during the current study are available from the corresponding author upon reasonable request.

## Abbreviations

CMR: Cardiac magnetic resonance imaging
COPD: Chronic obstructive pulmonary disease
CPET: Cardiopulmonary exercise test
CRF: Cardiorespiratory fitness
CVD: Cardiovascular disease
CT: Computed tomography
ECVF: Extracellular volume fraction
FACT-G7: Functional Assessment of Cancer Therapy-General
Gy: Gray units
HF: Heart failure
HR: Heart rate
LGE: Late-gadolinium enhancement
LVEDV: Left-ventricular end-diastolic volume
LVEF: Left-ventricular ejection fraction
LVESV: Left-ventricular end-systolic volume
MCRD: Mean cardiac radiation dose
NTproBNP: N-terminal pro-brain natriuretic peptide
O_2_: Oxygen
%O_2_Pulse: Percent-predicted peak oxygen pulse
RER: Respiratory exchange ratio
SpO_2_: Peripheral oxygen saturation
SV: Stroke volume
T1: Time constant representing the recovery of longitudinal magnetization
V: Volume
VO_2_: Oxygen consumption
