# Supplementary figures and images for: Influence of extracellular volume fraction on peak exercise oxygen pulse following thoracic radiotherapy
Source: Cardiooncology. 2022 Jan 18;8:1. doi: 10.1186/s40959-021-00127-6 (PMC8764840; doi:10.1186/s40959-021-00127-6)

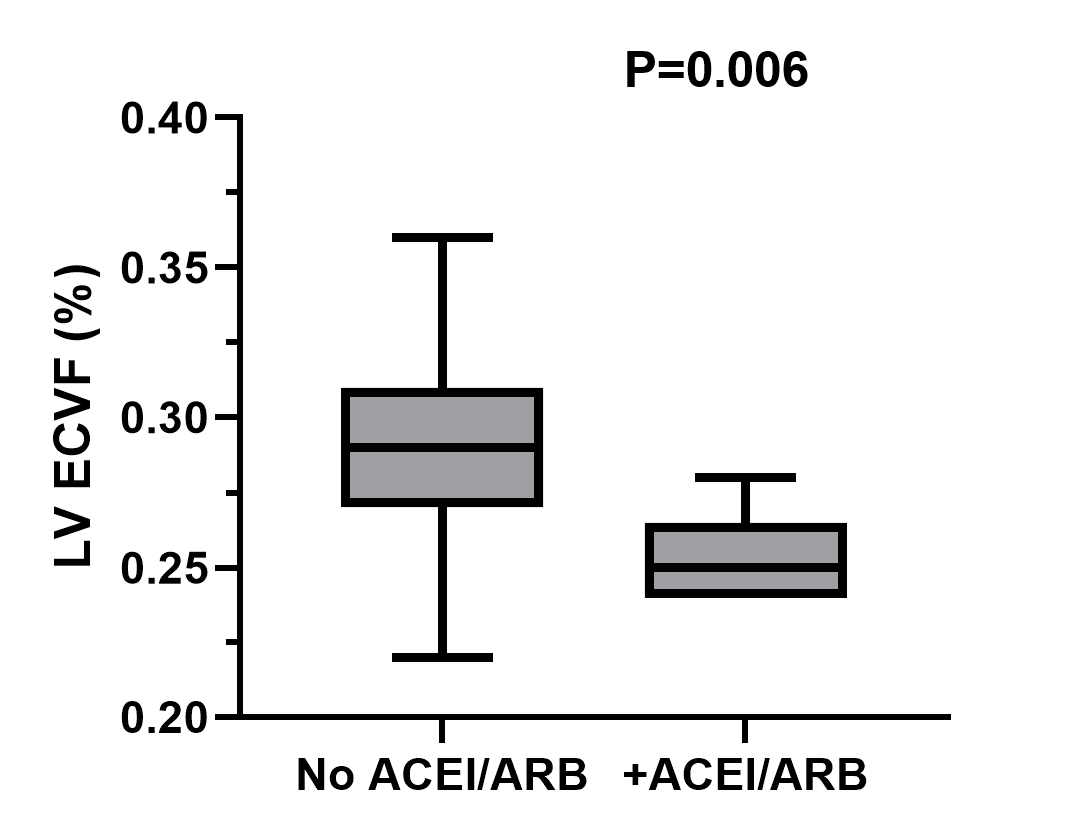

Supplement: Supplementary file 1 — Additional file 1. [file 40959_2021_127_MOESM1_ESM.tif]
