# Supplementary material for: Influence of extracellular volume fraction on peak exercise oxygen pulse following thoracic radiotherapy
Source: Cardiooncology. 2022 Jan 18;8:1. doi: 10.1186/s40959-021-00127-6 (PMC8764840; doi:10.1186/s40959-021-00127-6)
Supplement: Supplementary file 2 — Additional file 2. [file 40959_2021_127_MOESM2_ESM.docx]

| Supplemental Table 1. Chemotherapy Regimens of the Cohort. | | |
| --- | --- | --- |
| **Prior Chemotherapy Use** | **N (%)** | **Dose** |
| Paclitaxel | 18 (60%) | 483 ± 289 mg/m^2^ |
| Carboplatin | 13 (43%) | 863 ± 636 mg |
| Cyclophosphamide | 8 (27%) | 2540 ± 523 mg/m^2^ |
| Doxorubicin | 7 (23%) | 234 ± 44 mg/m^2^ |
| Cisplatin | 5 (17%) | 271 ± 82 mg |
| Etoposide | 4 (13%) | 597 ± 248 mg/m^2^ |
| Pemetrexed | 2 (7%) | 1500 ± 707 mg/m^2^ |
| Imatinib | 1 (3%) | 300 mg |
| Rituximab | 1 (3%) | 375 mg/m^2^ every 3-months |
| Nivolumab | 1 (3%) | 2240 mg |
| Trastuzumab | 1 (3%) | 104 mg/kg |
| Pertuzumab | 1 (3%) | 1260 mg/kg |
| Vinblastine | 1 (3%) | 36 mg/m^2^ |
| Dacarbazine | 1 (3%) | 2250 mg/m^2^ |
| Bleomycin | 1 (3%) | 60 u/m^2^ |
| Data are listed as n (%) and mean ± standard deviation. | | |
