# Supplementary material for: Influence of extracellular volume fraction on peak exercise oxygen pulse following thoracic radiotherapy
Source: Cardiooncology. 2022 Jan 18;8:1. doi: 10.1186/s40959-021-00127-6 (PMC8764840; doi:10.1186/s40959-021-00127-6)
Supplement: Supplementary file 3 — Additional file 3. [file 40959_2021_127_MOESM3_ESM.docx]

| Supplemental Table 2. Clinical Characteristics of the Cohort divided by the median ECVF%; N=27 | | | |
| --- | --- | --- | --- |
| Variables | ECVF ≤28%  n=15 | ECVF >28%  n=12 | *P*-Value |
| Age, y | 63 [59-73] | 64 [58-64] | 0.378 |
| Female | 11 (73%) | 4 (33%) | 0.038 |
| Caucasian | 10 (67%) | 8 (67%) | 1.000 |
| Body Mass Index, kg/m^2^ | 28.2 [25.1-30.8] | 26.5 [20.8-30.9] | 0.382 |
| COPD | 5 | 8 | 0.412 |
| FEV_1_, % | 92 [73-102] | 64 [44-76] | 0.016 |
| FACT-G7 score | 21.0 [17.8-25.0] | 17.0 [14.0-22.5] | 0.042 |
| Cancer type |  |  | 0.014 |
| Lung or Other* | 7 (47%) | 11 (92%) |  |
| Breast | 8 (53%) | 1 (8%) |  |
| Time since Diagnosis, y | 2.1 [0.7-3.0] | 3.2 [1.6-7.9] | 0.064 |
| Time since Radiotherapy, y | 1.5 [0.2-2.6] | 2.9 [0.8-6.7] | 0.157 |
| Time since chemotherapy, y | 1.7 [0.5-2.7] | 2.3 [0.8-6.6] | 0.273 |
| MCRD, Gy | 5.4 [3.6-10.7] | 12.2 [4.36-19.7] | 0.236 |
| Prior Chemotherapy | 12 | 12 | 0.100 |
| CVD Risk Factors |  |  |  |
| Hypertension | 10 (67%) | 5 (42%) | 0.194 |
| Diabetes Mellitus | 5 (33%) | 2 (17%) | 0.326 |
| Hypercholesterolemia | 8 (53%) | 4 (33%) | 0.299 |
| Current Smoker | 2 (13%) | 3 (25%) | 0.438 |
| Obesity | 5 (33%) | 4 (33%) | 1.000 |
| Beta-blocker Use | 2 (13%) | 3 (25%) | 0.438 |
| ACE-I/ARB | 6 (40%) | 0 (0%) | 0.013 |
| NTproBNP, pg/mL | 59 [34-245] | 225 [166-406] | 0.079 |
| VO_2_, mL·kg^-1^·min^-1^ | 19.6 [15.7-22.6] | 16.5 [13.1-18.6] | 0.067 |
| %O_2_ Pulse | 93 [80-111] | 66 [53-84] | 0.002 |
| CMR Parameters |  |  |  |
| LVEF, % | 70 [64-75] | 59 [49-64] | 0.016 |
| Cardiac Index, L/min/m^2^ | 2.6 [2.2-2.9] | 2.8 [1.8-3.0] | 1.000 |
| SVI, mL/m^2^ | 40 [34-45] | 31 [28-43] | 0.164 |
| Presence of LGE | 6 (40%) | 5 (42%) | 0.930 |
| LV ECVF-Global, % | 26 [24-27] | 31 [29-32] | <0.001 |
| Native T1-Global, ms | 1027 [991-1048] | 1047 [1029-1091] | 0.028 |
| Post-contrast T1-Global, ms | 434 [416-461] | 433 [389-470] | 0.826 |
| Data are listed as median and [interquartile range] or n (%). *Other malignancies grouped with lung cancer subjects. *P*-values are differences between groups (≤28% and >28% ECVF).  Abbreviations: ACE-I/ARB=angiotensin converting enzyme-inhibitor/angiotensin receptor blocker; CI=cardiac index; COPD=chronic obstructive pulmonary disease; CVD=cardiovascular disease; ECVF= extracellular volume fraction; FACT-G7= Functional Assessment of Cancer Therapy-General (7-item version); FEV_1_=forced expiratory volume 1-second; LV ECVF=left-ventricular extracellular volume fraction; LVEF=left-ventricular ejection fraction; MCRD=mean cardiac radiation dose; NTproBNP=N-terminal pro-brain natriuretic peptide. | | | |
